# Supplementary figures and images for: Dissecting infant leukemia developmental origins with a hemogenic gastruloid model
Source: eLife. 2025 Sep 11;14:RP102324. doi: 10.7554/eLife.102324 (PMC12425479; doi:10.7554/eLife.102324)

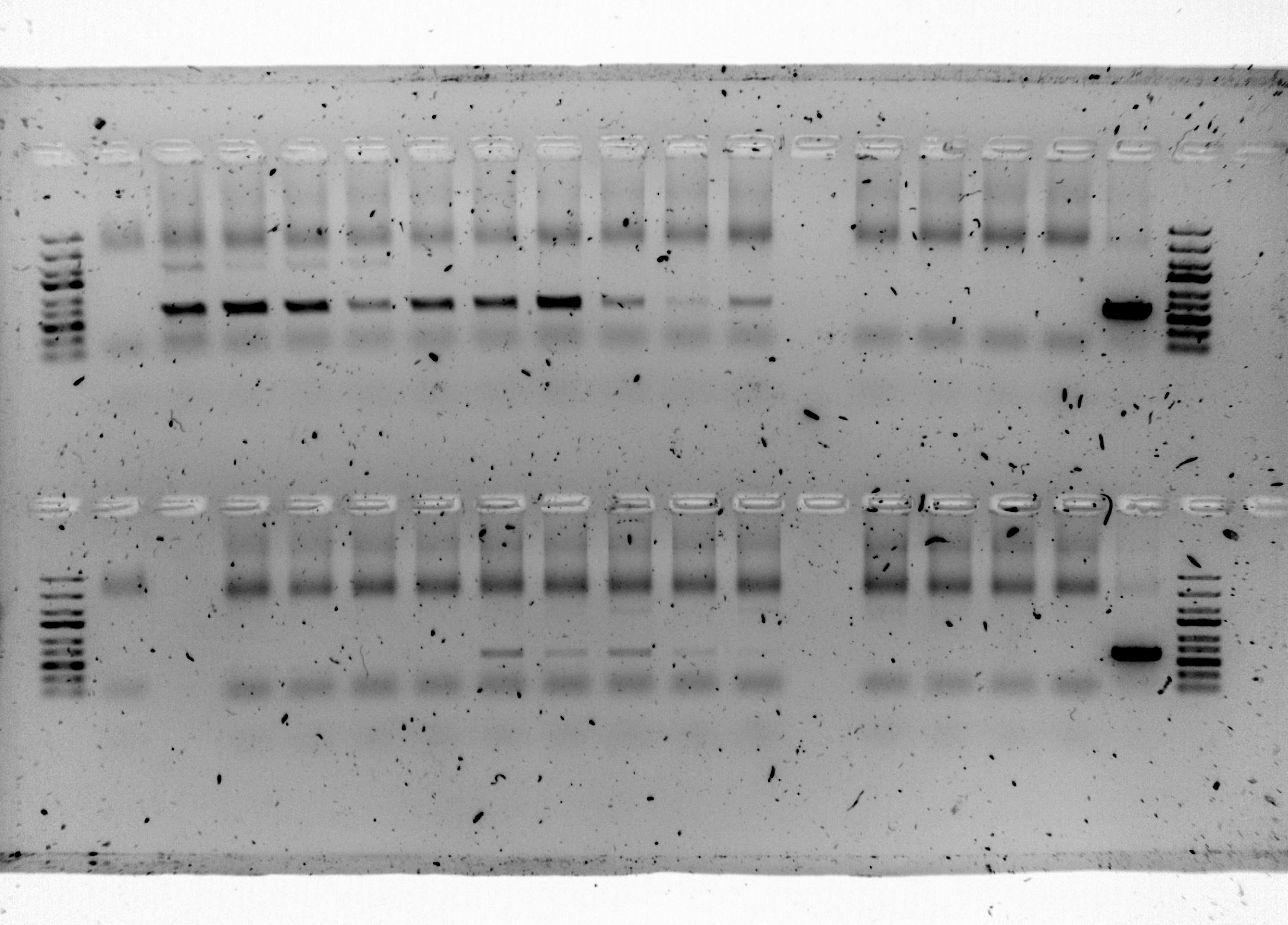

Supplement: Figure 4—source data 1. [file elife-102324-fig4-data1.zip › Fig_4C_BM.tif]

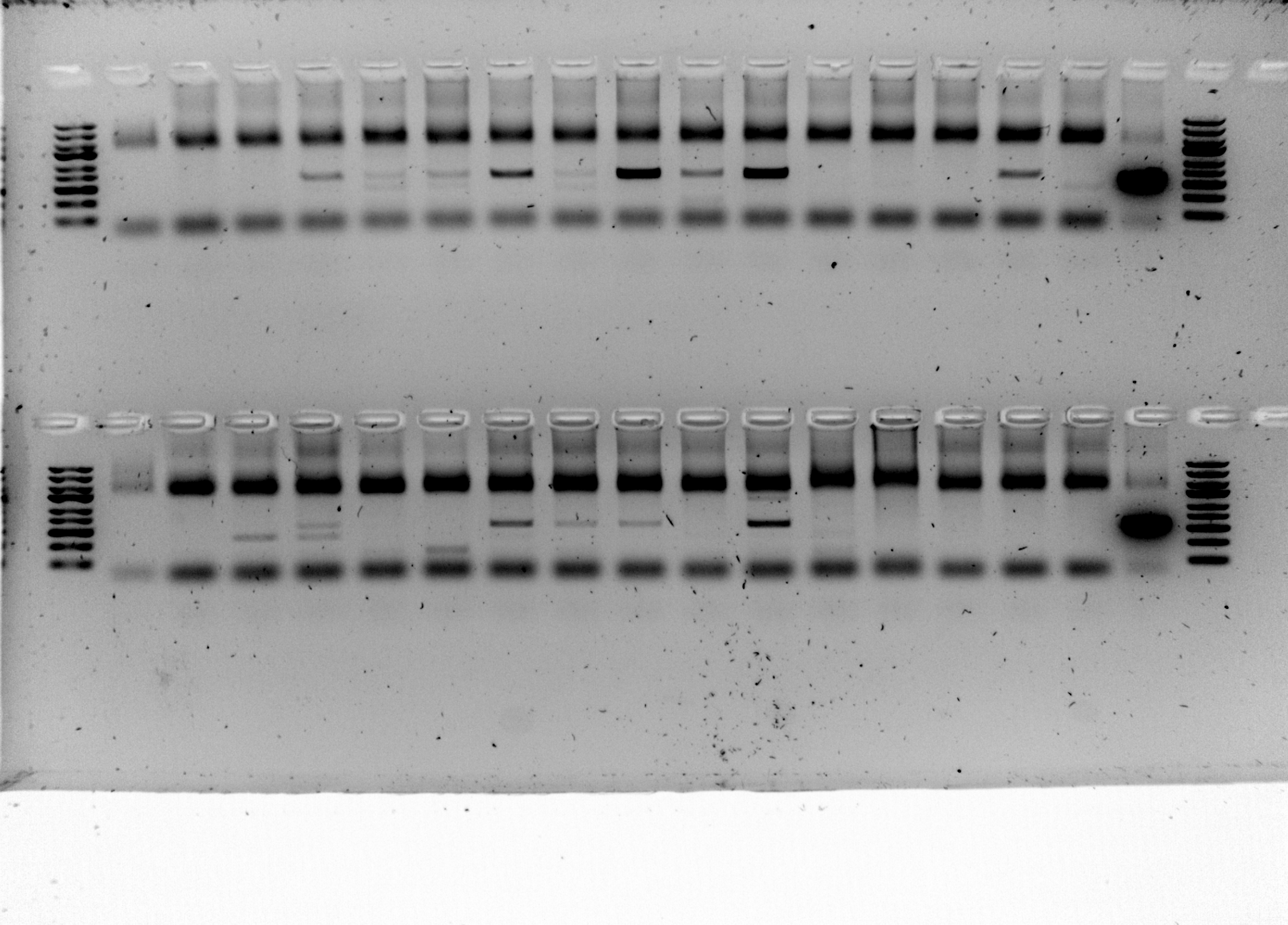

Supplement: Figure 4—source data 1. [file elife-102324-fig4-data1.zip › Fig_4C_Spl.tif]

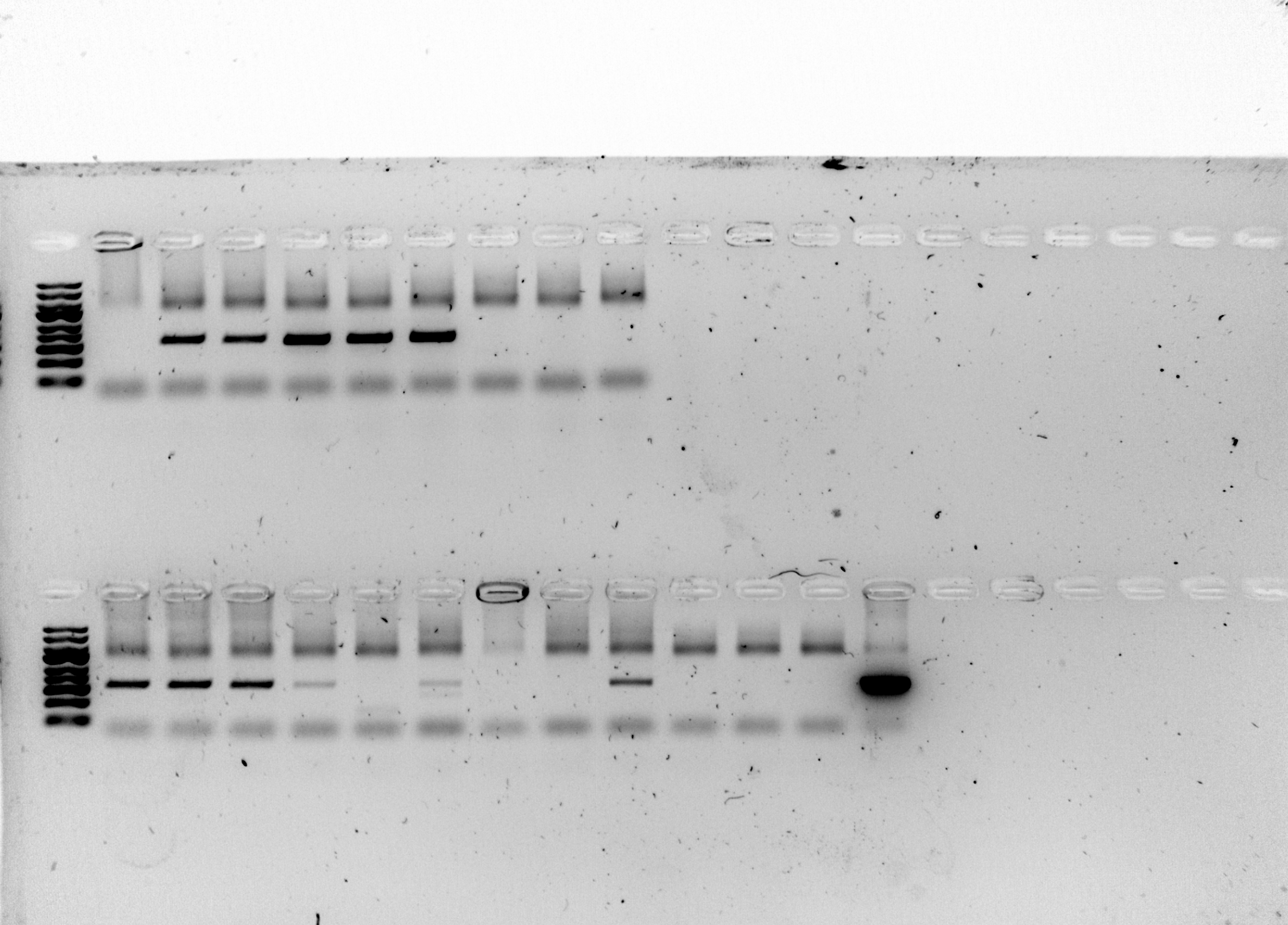

Supplement: Figure 4—figure supplement 2—source data 1. [file elife-102324-fig4-figsupp2-data1.zip › Fig_4S2_E.tif]

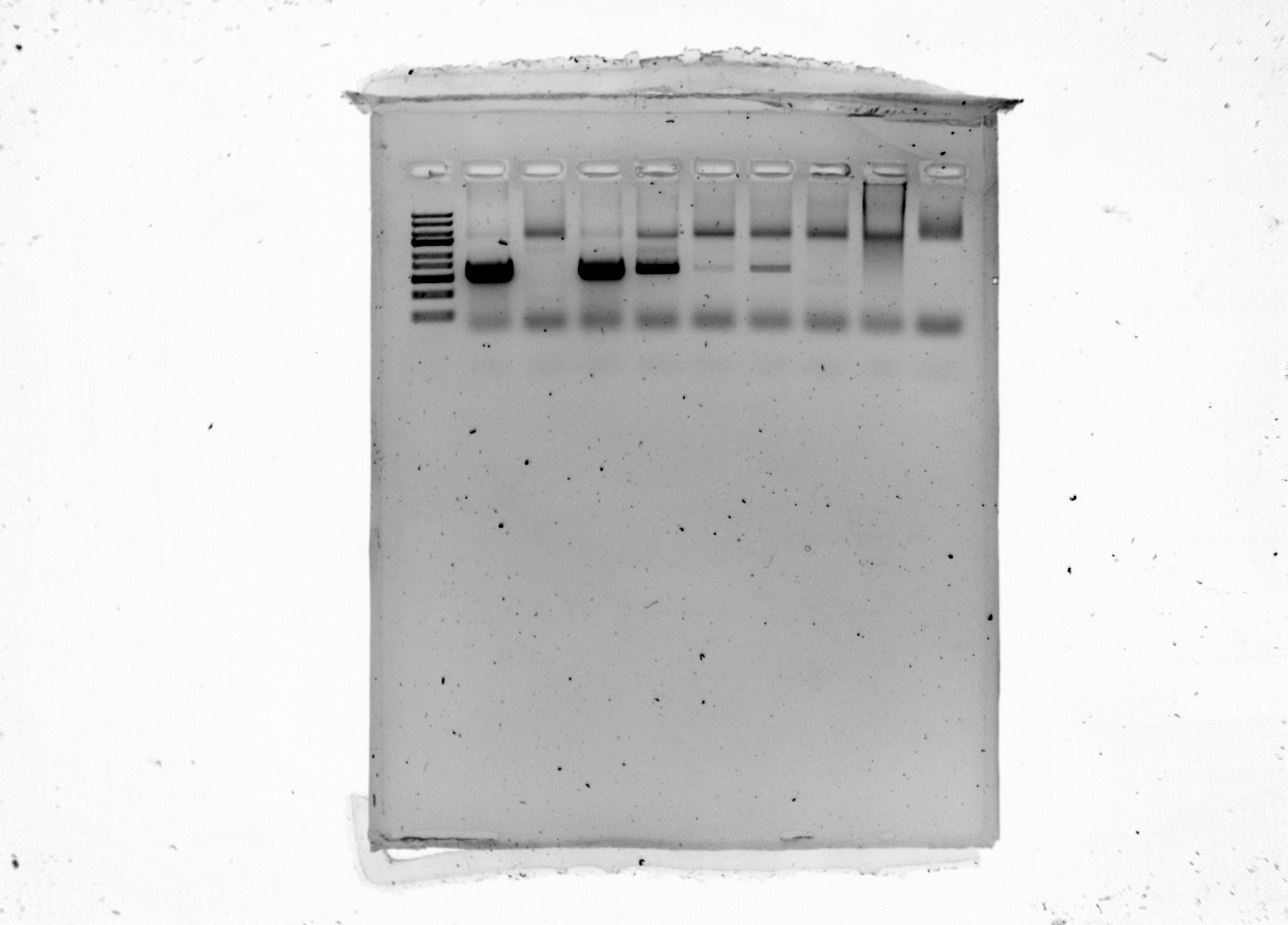

Supplement: Figure 4—figure supplement 2—source data 1. [file elife-102324-fig4-figsupp2-data1.zip › Fig_4S2_B.tif]
