# Supplementary material for: Dissecting infant leukemia developmental origins with a hemogenic gastruloid model
Source: eLife. 2025 Sep 11;14:RP102324. doi: 10.7554/eLife.102324 (PMC12425479; doi:10.7554/eLife.102324)
Supplement: Figure 4—source data 2. [file elife-102324-fig4-data2.zip › Figure 4 - source data 2.pdf]

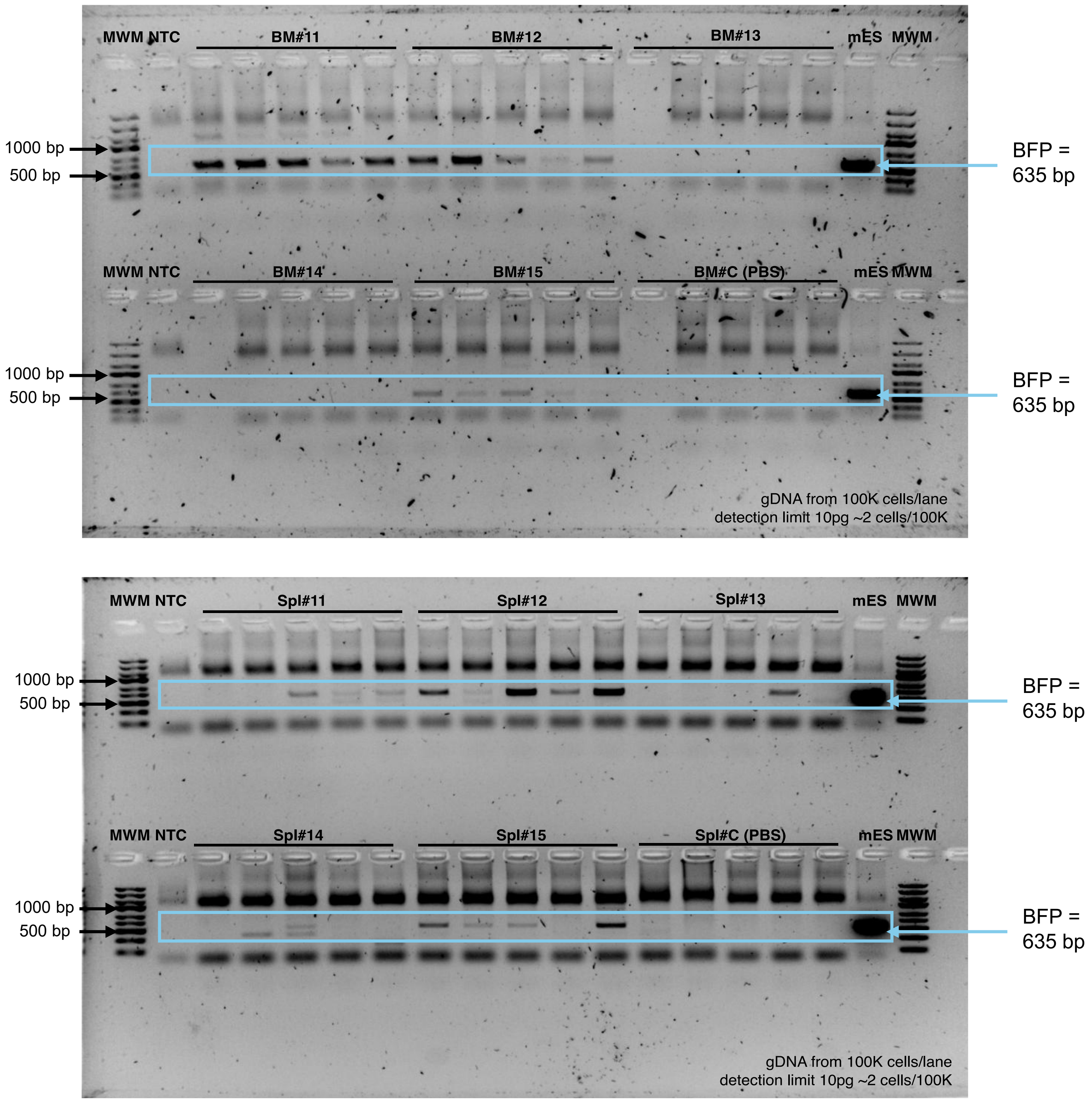

Figure 4 – source data

Uncropped gel electrophoresis corresponding to Figure 4C – top : bone marrow (BM); bottom : spleen (Spl). MWM : molecular weight marker.

PCR detection of haemGx genomic (g)DNA in the bone marrow (BM) and spleen (Spl) of immunodeficient mice 4 weeks after unilateral adrenal implantation of 3 gastruloids/gland. Analysis of 5 replicates of 100ng gDNA/recipient tissue; control animal was injected unilaterally with PBS in an adrenal gland in parallel with experimental implantation. Reaction positive control used 100ng of gDNA from *Rosa26-BFP*::*Flkj1-GFP* mES cells (mES) used to generate haemGxs. NTC: no template control.
